# A neuromuscular respiratory outpatient clinic: Patient profile and experience (Beaumont Hospital)

**DOI:** 10.1186/1753-6561-9-S1-A28

**Published:** 2015-01-14

**Authors:** K Carty, M Guidon, C Egan

**Affiliations:** 1 Department of Physiotherapy, Royal College of Surgeons in Ireland, 123 St. Stephen’s Green, Dublin 2. Ireland; 2 Beaumont Hospital, Dublin 9, Ireland

## Background

Neuromuscular disorders (NMD) such as Duchenne Muscular Dystrophy (DMD) are characterised by slow progressive muscle atrophy and weakness [1]. Weak respiratory muscles, loss of ambulation and mechanical factors, cause a reduction in total lung capacity (TLC) and Vital Capacity (VC). Patients are at risk of developing acute conditions that decrease pulmonary function and in turn trigger acute respiratory failure [2]. Due to advancements in respiratory interventions NMD patients are progressing into an adulthood healthcare setting, there is a need for intervention in this phase of transition [1]. Currently in Ireland, children with NMD are diagnosed in a paediatric setting and transition to adult services without a designated medical consultant. In January 2012 a multidisciplinary Neuromuscular Respiratory Clinic was established in Beaumont Hospital. This is the first clinic of its kind in an adult setting in Ireland.

## Methods

The main aim of this project was to profile the patients who have attended the clinic since its establishment and to survey all patients who attended to determine patient clinic satisfaction. The patient profile was completed using a retrospective review of patient charts that attended the clinic from January 2012. Patients were surveyed at a clinic visit during the summer period.

## Results

Forty-one patient records were reviewed. The majority of the patients (56%) presented with a form of Muscular Dystrophy (MD). 76% of patients were male. The age range upon first visit was 18 – 69 years with 51% of patients under 35yrs. 78% of patients reported a Peak Cough Flow below 270 L/min and 22% had an FVC of less than 1 Litre. 7 out of the 13 patients who had pulmonary function tests carried out recorded an FEV1 less than 50% of the predicted value. Interventions provided included breath stacking, deep breathing exercises and the use of a cough assist device. Eleven patients made a return visit to the clinic within one year of their first appointment. Due to unforeseen circumstances only eight patients completed the survey, 88% of patients felt they could had a good communication with the care team and 50% felt their visit to the clinic would lead to fewer health problems.

## Conclusions

The low age profile justifies the need for this clinic. There is currently no clinic in Ireland accommodating this patient group and access to a care-team such as this is vital to the continued management of their condition. Overall patient satisfaction was positive although the number surveyed was small.
